# Prevalence and Risk Factors of Schistosomiasis in Sudan: A Systematic Review and Meta-Analysis

**DOI:** 10.7759/cureus.73966

**Published:** 2024-11-19

**Authors:** Yousef Alsaafin, Ayman Omer, Osama Felemban, Sarra Modawi, Maydolin Ibrahim, Abdullah Mohammed, Ammar Elfaki, Ahmed Abushara, Maryam A SalahEldin

**Affiliations:** 1 Internal Medicine, Dr. Soliman Fakeeh Hospital, Riyadh, SAU; 2 Gastroenterology and Hepatology, Prince Sultan Military Medical City, Riyadh, SAU; 3 Emergency, Security Forces Hospital, Riyadh, SAU; 4 Internal Medicine, Specialized Medical Center Hospital, Riyadh, SAU; 5 Medical Microbiology, University of Khartoum, Khartoum, SDN

**Keywords:** africa, communicable diseases, developing countries, intestinal parasite, middle east

## Abstract

Schistosomiasis is considered the most widespread parasitic infection. Both *Schistosoma haematobium* and *Schistosoma mansoni* are present, and as waterborne infections, their epidemiology is closely associated with proximity and exposure to freshwater sources. The objective of the current study is to estimate the pooled prevalence of schistosomiasis among the Sudanese population and examine any associated sociocultural risk factors. A systematic review was conducted in December 2022. The review was conducted in accordance with the Preferred Reporting Items for Systematic Reviews and Meta-Analyses (PRISMA) guidelines. Sixty-seven research articles were recruited representing a total sample size of 813,571 participants. *Schistosoma haematobium* pooled prevalence was 24.83% (95% confidence interval (CI): 22.75, 26.92) among 700,337 participants tested, while *S. mansoni* pooled prevalence of 19.13% (95% CI: 18.70, 19.56) among 685,133 participants was originated. Moreover, schistosomiasis prevalence among school-age children was assessed in 45 included studies; the pooled prevalence of *S. haematobium* was 22.37 (95% CI: 20.12, 24.63), while* S. mansoni* pooled prevalence was 18.62 (95% CI: 13.14, 24.11). Furthermore, the highest *Schistosoma* prevalence (overall pooled prevalence: 41% (95% CI: 26.72, 55,29), *S. haematobium* pooled prevalence: 38.59 (95% CI: 21.03, 56.14), *S. mansoni* pooled prevalence: 25.85 (95% CI: 5.07, 46.63)) was found among Gezira State participants, based on a sample size of 5,712 individuals. Farming, male gender, no presence of latrines, canal and stream water sources, and swimming, playing, or bathing in the Nile River and canals were found to be significantly associated with schistosomiasis infection. The current findings are believed to serve as a cornerstone for designing strategies and preventive measures.

## Introduction and background

Considering the ongoing political turmoil, marked by decades of war and hostility in Sudan, healthcare has largely been neglected, overshadowed by what the government may deem as more urgent concerns. The country is confronting a worsening humanitarian crisis, with almost eight million people facing severe challenges to their psychological and clinical well-being, including approximately 1.6 million internally displaced individuals and around one million refugees. Resources are limited, and the country's economic output dropped by almost 67% between 2017 and 2018, even before the current armed conflict. Healthcare infrastructure is inadequately resourced and unable to meet the increasing and neglected demands. To make matters worse, Sudan remains far from achieving the Sustainable Development Goals (SDGs). These sociopolitical and economic challenges may increase vulnerability to infectious diseases by disrupting healthcare infrastructure, limiting access to healthcare services, and creating conditions conducive to disease transmission. The primary communicable diseases contributing to morbidity in the country include malaria, tuberculosis, schistosomiasis, pneumonia, and diarrheal diseases, according to the WHO and the Sudan Health Observatory under the Federal Ministry of Health [1,2].

Schistosomiasis is recognized as the most common parasitic infection. Both *Schistosoma haematobium* and *Schistosoma mansoni* are present in the region, and as waterborne diseases, their distribution is closely linked to the availability and accessibility of natural freshwater sources. A recent nationwide survey involving over 100,000 school-age children has highlighted the widespread nature of this infection. The overall prevalence of* S. haematobium* was found to be 5.2%, while *S. mansoni* showed a prevalence of 0.06%. However, other studies have reported even higher localized prevalence rates; for instance, in certain schools within White Nile State, 46.5% of the children sampled were infected, with 45% infected with* S. haematobium*, 5.9% with *S. mansoni*, and 4.4% with mixed infections [3]. This study aims to estimate the pooled prevalence of schistosomiasis among the Sudanese population and identify related social and cultural risk factors. This objective is crucial due to the variability and limited scope of existing studies, which are often region-specific and hinder a cohesive understanding of national prevalence. By synthesizing diverse data, this study provides a reliable, comprehensive estimate and highlights sociocultural risk factors, contributing valuable insights to guide targeted, equitable disease control strategies across Sudan.

## Review

Materials and methods

Search Strategy

To identify relevant studies, a systematic review of the literature was conducted in December 2022. The review was regulated in accordance with the Preferred Reporting Items for Systematic Reviews and Meta-Analyses (PRISMA) statement [4]. A comprehensive search was conducted across Google Scholar, Scopus, PubMed, Embase, Directory of Open Access Journals (DOAJ), Index Copernicus, Elton B. Stephens Company (EBSCO)-Cumulative Index to Nursing and Allied Health Literature (CINAHL), and Cochrane databases, without language restrictions (although studies in languages other than English were later excluded). To ensure relevance to the current situation, only studies published from 2010 onward were included. Additionally, studies with data collection prior to 2010 were excluded, except where data collection began in or before 2010 and continued into 2010 or beyond, as previously described [5].

Due to the limited availability of medical literature from Sudan in international databases and the variability in the reporting of sociocultural factors, these factors were not included in the formulation of keywords. Instead, relevant information was extracted from the studies that were included later on. The keywords used in PubMed were as follows: "Schistosomiasis" OR "Schistosoma mansoni" OR "Schistosoma haematobium" OR "Schistosoma japonicum" AND "Sudan*[tiab]", as previously described [6].

Furthermore, to refine the search process, manual searches of the reference lists from the included articles were conducted.

Study Selection and Data Extraction

Titles and abstracts were evaluated for initial eligibility. Full texts were obtained for all research articles that were available and tentatively approved for inclusion. Data abstraction followed a task separation approach; the methods and results sections of each study were abstracted separately on different occasions to minimize bias. Additionally, abstraction was performed without regard to the authors' qualifications or expertise. All authors carefully selected relevant studies from the literature, and any disagreements that arose during the process were resolved through thorough discussion and consensus. Each research article was examined for all pertinent information and recorded in a data extraction file (Microsoft Excel, Microsoft Corp., Redmond, WA). Data from each methods section were extracted using a predefined set of variables, including study characteristics, participant demographics, study population size, geographical region, methodology employed for prevalence or risk assessment, and study duration. Moreover, since risk factor-related keywords were not included in the search strategy, each study was thoroughly screened to identify the nature of the risks investigated. Studies that did not assess prevalence or sociocultural risks were subsequently excluded, as previously described [5].

Assessment of Quality and Risk of Bias

Each article included in the review was assessed using a structured framework designed for summarizing quality evaluations. The existing literature was examined, and a specific framework was developed to evaluate the representativeness of the studied population and assess the strength of the estimates reported. Each article was required to address five questions, with responses scored as follows: 1 point for "yes," 0 points for "no," and 0 points for "not available." The total score for risk of bias and quality was calculated by summing the scores across all five domains, yielding a score ranging from 0 to 5. A higher score indicates superior quality, and only studies with a quality score of 3 or above were included in the analysis, as previously described [5].

As outlined previously [5], the five criteria evaluated were as follows: is the study objective explicitly stated, is the study population well-defined and specified, is the study sample comprehensively identified, is the methodology robust, and is the data analysis robust?

Secondary Analysis

Among all the included studies that reported either prevalence or risk factor estimates, it was noted whether the standard error (SE) was provided. For studies that did not report the SE, it was calculated using the following formula: SE = √p (1-p)/n, where p represents prevalence. Regarding risk factors, each included study may have had different objectives, which influenced how results were presented (e.g., adjusted odds ratio (OR), unadjusted OR, or frequencies). For each sociocultural variable investigated, the odds ratio (OR) was calculated for individual categories whenever possible, allowing for univariate analysis of each category within the studied population, as previously described [5].

The categorization of variables was structured to enhance the population size for specific estimates. For instance, while most studies examining the sociocultural risks of schistosomiasis classified education levels as below secondary and secondary/above, the few studies that used a primary, secondary, and university classification were re-categorized to combine similar groups. This resulted in a new classification where "primary" was defined as below secondary, and "secondary and university" were combined into the secondary/above category, as previously described [5].

Quantitative Analysis

Meta-analysis was conducted using Review Manager software versions 5.3 and 5.4 (The Cochrane Collaboration, London, UK) whenever feasible. The software automatically calculated the confidence interval (CI) based on the provided standard error (SE), and if a CI was reported in a study, it was incorporated accordingly. The heterogeneity of each meta-analysis was also evaluated, with the random effects model preferred over the fixed effects model due to the expected variability between study populations. Sensitivity analysis was performed to assess the impact of studies conducted in populations thought to behave similarly or presumed to have low risk on the overall pooled data. Additionally, subgroup analyses were carried out when appropriate to determine prevalence or risk levels within specific states or populations. An outcome needed to be included in at least two studies to be considered for the meta-analysis. The trim-and-fill method was employed to evaluate the risk of publication bias in each meta-analysis performed, as previously described [5,7].

Results

Studies Included

**Figure 1:**
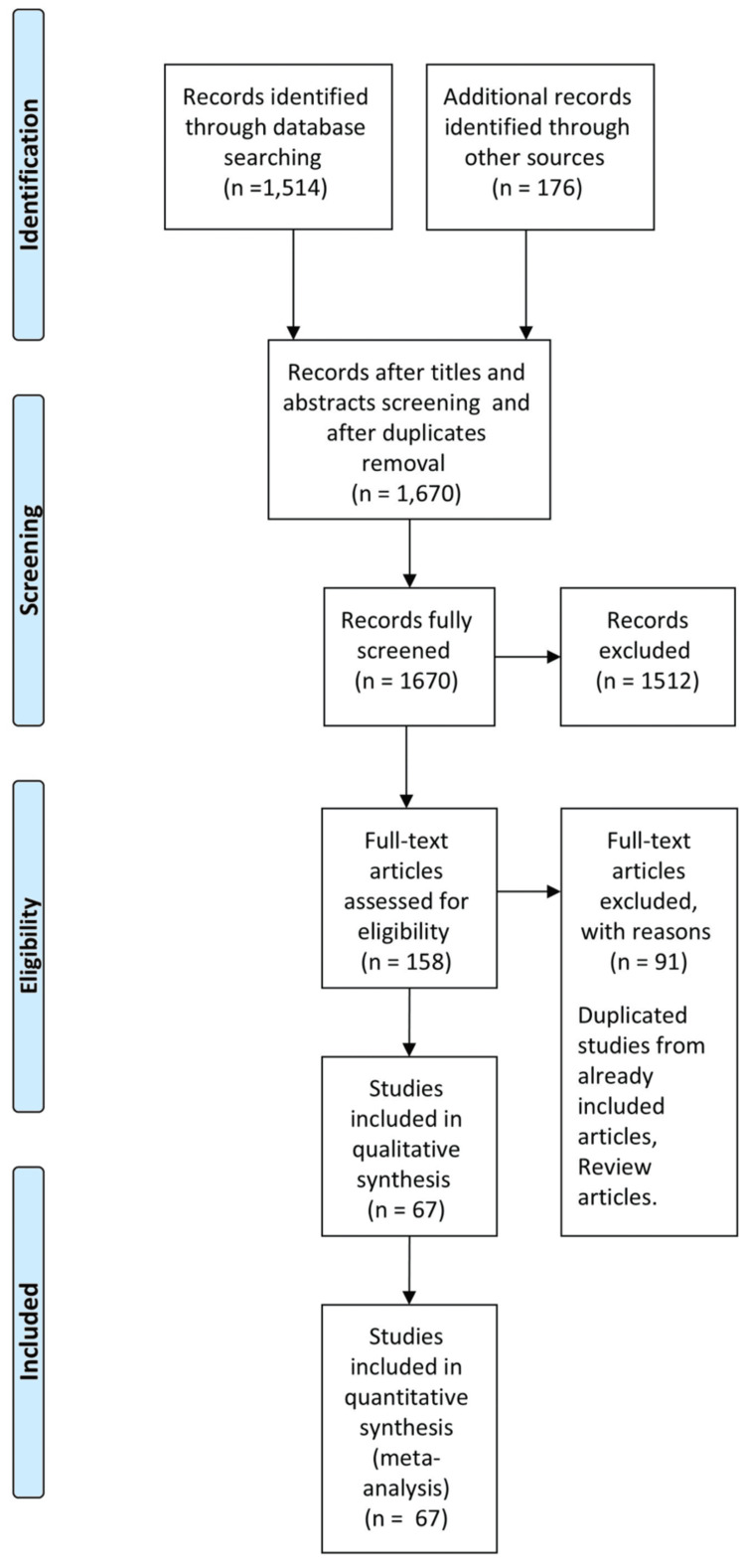
PRISMA flow diagram PRISMA: Preferred Reporting Items for Systematic Reviews and Meta-Analyses

A total of 1,690 articles were identified using the search strategy, which included manual searches of reference lists from pertinent original research articles and reviews. Out of these, 1,512 articles were excluded. Subsequently, after screening the abstracts and full texts, 67 articles met our inclusion criteria and successfully passed the quality assessment. These articles provided information on prevalence in specific populations and/or associated risk factors. The PRISMA flow diagram and checklist are shown in Figure 1 and Appendices, respectively. The quality assessment and risk of bias of included studies is provided in the Appendices.

Characteristics of the Studies

The characteristics of the included studies are outlined in Table 1. Sixty-seven research articles were recruited [8-74], among which 63 research articles determined the prevalence of schistosomiasis among different study populations. The earliest was published in 2010, while the most recent articles were published in 2022. Seventeen studies were conducted in White Nile State, 13 in Khartoum State, 11 in Gezira State, eight in Kassala State, four in River Nile State, three in Sennar State, two in southern Kordofan State, and one in each of Gadarif, Northern State, South Darfur, and both of Khartoum and Kassala States. Moreover, two studies were conducted among all 18 states of Sudan. All included studies represent a total sample size of 813,571 participants. Moreover, 53 articles were conducted among both genders, seven studies were conducted among only males, one study was conducted among only females, and the remaining three studies did not specify the gender of their participants. Moreover, the majority of studies (45) focused on the prevalence/sociocultural risk factors among school-age children; several studies included the general population, encompassing both school-age children and others; two studies were conducted among patients and suspected patients; one study focused on pregnant women; and another study was toward fishermen. Publication bias assessment indicated no major asymmetry.

**Table 1:** Characteristics of the included studies PCR: polymerase chain reaction, ELISA: enzyme-linked immunosorbent assay, IHA: indirect hemagglutination assay

Study ID	Publication year	Study design	State	Study population(s)	Assessment	Sample size	Gender	Participants' age (years)
Abakar et al. [8]	2021	Cross-sectional	Khartoum	Patients	Prevalence (parasitological methods) and risk factors	150	Both	5-≥35
Elfaki et al. [25]	2015	Cross-sectional	Kassala	General population	Prevalence (parasitological methods) and risk factors	100	Both	Mean: 19±13
Abdalla et al. [40]	2020	Cross-sectional	Khartoum	School-age children	Prevalence (parasitological methods) and risk factors	102	Not determined	6-20
Abdalla [42]	2013	Cross-sectional	White Nile	School-age children	Prevalence (parasitological methods) and risk factors	1,257	Not determined	5-19
Abdelgadir et al. [73]	2012	Cross-sectional	Gezira	Pregnant women	Prevalence (parasitological methods)	292	Females	Not determined
Abdelrhman et al. [9]	2017	Cross-sectional	White Nile	School-age children	Prevalence (parasitological methods) and risk factors	200	Both	6-≥15
Abdo et al. [59]	2015	Cross-sectional	Gezira	School-age children and general population	Prevalence (parasitological methods)	203	Males	10-55
Abou-Zeid et al. [18]	2012	Cross-sectional	Southern Kordofan	General population	Prevalence (parasitological methods) and risk factors	1,826	Both	Not determined
Abou-Zeid et al. [70]	2013	Cross-sectional	Southern Kordofan	School-age children	Prevalence (parasitological methods) and risk factors	2,302	Both	<8-≥12
Afifi et al. [51]	2016	Cross-sectional	Kassala	General population	Prevalence (parasitological methods) and risk factors	2,433	Both	1-≥50
Ahmed et al. [35]	2012	Cohort	Gezira	School-age children	Prevalence (parasitological methods) and risk factors	2,741	Both	6-15
Ahmed et al. [10]	2012	Cohort	Gezira	School-age children	Prevalence (parasitological methods) and risk factors	420	Both	1-16
Ahmed et al. [31]	2015	Cohort	Khartoum	Suspected patients	Prevalence (radiological methods)	109	Both	Mean: 58
Al-Basheer et al. [16]	2017	Cross-sectional	Khartoum	School-age children	Prevalence (parasitological methods) and risk factors	150	Males	<11-≥11
Alsanosi et al. [67]	2019	Cross-sectional	Khartoum	Children (general population)	Prevalence (parasitological methods) and risk factors	240	Both	≤16
Altijani et al. [63]	2017	Cross-sectional	White Nile	School-age children	Prevalence (parasitological methods)	182	Both	5-14
Amin et al. [48]	2017	Cross-sectional	Gezira	School-age children	Prevalence (parasitological methods)	500	Both	11-14
Bakhit et al. [54]	2019	Cross-sectional	White Nile	General population	Prevalence (parasitological methods)	1,029	Both	Mean: 15
Cha et al. [43]	2019	Cross-sectional	All 18 states of Sudan	School-age children	Prevalence (parasitological methods) and risk factors	105,167	Both	Not determined
Cha et al. [56]	2020	Cross-sectional	White Nile	School-age children	Prevalence (parasitological methods) and risk factors	2,784	Both	≤9-≥13
Deribe et al. [69]	2011	Cross-sectional	South Darfur	School-age children and general population	Prevalence (parasitological methods) and risk factors	811	Both	≤5->15
El-amin et al. [36]	2014	Cross-sectional	Gezira	School-age children	Prevalence (parasitological methods, radiological methods, and PCR)	438	Both	Mean: 11
Elbasheir et al. [58]	2020	Longitudinal survey	Sennar	School-age children	Prevalence (parasitological methods)	489	Both	5-15
Elfadol et al. [38]	2020	Cross-sectional	Khartoum	School-age children	Prevalence (parasitological methods) and risk factors	314	Both	7-18
Elfaki et al. [66]	2015	Cross-sectional	Kassala	General population	Prevalence (parasitological methods)	75	Both	Mean: 17
Elfaki et al. [37]	2015	Cross-sectional	Khartoum	General population	Prevalence (parasitological methods) and risk factor	141	Males	15-55
Elfaki et al. [64]	2016	Retrospective	Kassala	School-age children	Prevalence (parasitological methods and PCR) and risk factors	234	Both	4-85
Elfaki et al. [20]	2020	Cross-sectional	Khartoum	School-age children	Prevalence (parasitological methods) and risk factors	160	Both	Not determined
Elhag et al. [65]	2011	Cross-sectional	Gezira	General population	Prevalence (parasitological methods and ELISA) and risk factors	208	Both	4-80
Elmadani et al. [57]	2013	Cross-sectional	Gezira	School-age children	Prevalence (parasitological and radiological methods)	103	Males	7-20
Elmadhoun et al. [41]	2013	Cross-sectional	River Nile	School-age children	Prevalence (parasitological methods)	2,490	Both	8-19
Elmekki et al. [50]	2018	Cross-sectional	Khartoum and Kassala	School-age children	Prevalence (parasitological methods) and risk factors	770	both	4-85
Elsammani et al. [55]	2019	Cross-sectional	Khartoum	School-age children	Prevalence (parasitological methods)	600	Both	6-15
Elsiddig et al. [14]	2019	Cross-sectional	White Nile	School-age children	Prevalence (parasitological methods) and risk factors	385	Both	6-15
Gasmelseed et al. [44]	2012	Cross-sectional	Gezira	School-age children	Prevalence (parasitological and radiological methods)	438	Both	6-20
Gasmelseed et al. [26]	2014	Cross-sectional	Gezira	School-age children	Prevalence (parasitological and radiological methods, and PCR)	83	Males	6-20
Hajissa et al. [33]	2018	Cross-sectional	Khartoum	School-age children	Prevalence (parasitological methods) and risk factors	170	Both	6-17
Hamad et al. [46]	2018	Cross-sectional	River Nile	School-age children	Prevalence (parasitological methods)	200	Not determined	Not determined
Hassan et al. [22]	2019	Cross-sectional	Khartoum	School-age children	Prevalence (parasitological methods) and risk factors	134	Both	6-14
Ibrahim et al. [21]	2014	Cross-sectional	Sennar	School-age children	Prevalence (parasitological methods, ELISA, and IHA)	214	Both	6-16
Ibrahim et al. [62]	2019	Cross-sectional	Sennar	School-age children	Prevalence (parasitological methods)	396	Both	9-16
Ismail et al. [49]	2014	Cross-sectional	White Nile	School-age children	Prevalence (parasitological methods) and risk factors	338	Both	7-15
Jin et al. [27]	2022	Cross-sectional	All 18 states of Sudan	School-age children	Prevalence (parasitological methods) and risk factors	105,167	Both	Mean: 11
Jin et al. [19]	2020	Cohort	White Nile	School-age children	Prevalence (parasitological methods)	1,286	Both	6-16
Jin et al. [29]	2021	Cohort	White Nile	School-age children	Prevalence (parasitological methods)	1,951	Both	Mean: 9
Kardaman et al. [74]	2017	Cross-sectional	Gezira	School-age children	Prevalence (parasitological methods)	286	Both	3-14
Kassar [39]	2017	Cross-sectional	North Kordofan	School-age children	Risk factors	310	Both	8-16
Kebayer et al. [34]	2022	Cross-sectional	Kassala	General population	Prevalence (parasitological methods) and risk factors	190	Both	1-99
Khalid et al. [13]	2012	Cross-sectional	Gezira	Pregnant women	Risk factors	292	Female	Not determined
Kim et al. [11]	2016	Cross-sectional	White Nile	School-age children and general population	Prevalence (parasitological and radiological methods)	1,462	Both	1-80
Lee et al. [23]	2015	Cross-sectional	White Nile	School-age children and general population	Prevalence (parasitological methods)	561,517	Both	Not determined
Lee et al. [45]	2019	Cross-sectional	White Nile	General population	Prevalence (parasitological methods) and risk factors	1,138	Both	0-<30
Mahgoub et al. [60]	2010	Cross-sectional	Kassala	School-age children	Prevalence (parasitological methods) and risk factors	640	Both	8-18
Mahmood [12]	2016	Case-control	Khartoum	School-age children	Risk factors	768	Both	8-15
Malik et al. [61]	2021	Case-control	White Nile	Fishermen	Prevalence (parasitological methods, ELISA, and immunological assays)	119	Males	14-77
Mohamed et al. [15]	2013	Cross-sectional	Kassala	General population	Prevalence (parasitological methods)	770	Both	4-85
Mohammed et al. [17]	2018	Cross-sectional	White Nile	School-age children	Prevalence (parasitological methods) and risk factors	475	Both	6-15
Omer et al. [71]	2020	Cross-sectional	River Nile	School-age children	Prevalence (parasitological methods) and risk factors	1,188	Both	6-18
Osman et al. [28]	2018	Cross-sectional	Khartoum	School-age children	Prevalence (parasitological methods)	300	Both	5-13
Osman et al. [68]	2022	Cross-sectional	Northern State	School-age children	Prevalence (parasitological methods)	1,557	Males	6-13
Salah et al. [52]	2014	Cross-sectional	Gedarif	School-age children	Prevalence (parasitological methods) and risk factors	480	Both	Mean: 18
Sulieman et al. [30]	2017	Cross-sectional	River Nile	School-age children	Prevalence (parasitological methods) and risk factors	385	Both	7-≥14
Suliman et al. [72]	2021	Cross-sectional	White Nile	School-age children	Prevalence (parasitological methods) and risk factors	347	Both	10-17
Taha et al. [47]	2019	Cross-sectional	Khartoum	School-age children	Prevalence (parasitological methods)	1,205	Both	6-14
Talab et al. [24]	2018	Cross-sectional	White Nile	School-age children	Risk factors	420	Both	9-17
Tamomh et al. [32]	2018	Cross-sectional	White Nile	School-age children	Prevalence (parasitological methods) and risk factors	480	Both	5-≥12
Tamomh et al. [53]	2018	Cross-sectional	White Nile	School-age children	Risk factors	480	Both	5-≥12

Schistosomiasis Prevalence

Prevalence estimates were compiled to highlight the overall disease burden and assess the burden within specific subgroups based on the study population, causative agent, and geographic location, whenever feasible. Detailed pooled prevalence data is provided below, with a summary in Table 2.

**Table 2:** Summary of prevalence estimates synthesized from the included studies CI: confidence interval

Prevalence	Assessed in (state)	Assessed among	Total sample size	Pooled prevalence	95% CI
Prevalence of schistosomiasis	All 18 states of Sudan	General population, school-age children, suspected patients, farmers, pregnant women, and fishermen	812,801	26.86	24.71, 29.02
Prevalence of S. haematobium	Khartoum, Gezira, River Nile, Sennar, Gadarif, Northern State, South Darfur, and Kassala	School-age children, general population, suspected patients, and fishermen	700,337	24.83%	22.75, 26.92
Prevalence of S. mansoni	All 18 states of Sudan	General population, school-age children, and pregnant women	685,133	19.13	18.70, 19.56
Prevalence among the general population	White Nile, Khartoum, Gezira, Kassala, River Nile, Sennar, Southern Kordofan, Gadarif, Northern State, and South Darfur	General population	812,131	25.75	23.53, 27.97
Prevalence among school-age children	White Nile, Khartoum, Gezira, Sennar, River Nile, Kassala State, Gadarif, Southern Kordofan, and Northern State	School-age children	240,228	24.46%	22.78, 26.13
Prevalence in Khartoum State	Khartoum	School-age children, general population, and suspected patients	3,775	20.66%	11.74, 29.57
Prevalence in Gezira State	Gezira	Pregnant women, students, and general population	5,712	41.00%	26.72, 55.29
Prevalence in Kassala State	Kassala	General population and school-age children	5,212	30.33%	19.15, 41.51
Prevalence in River Nile State	River Nile	School-age children	4,263	17.33%	6.44, 28.22
Prevalence in Sennar state	Sennar	School-age children	1,099	28.60%	20.52, 36.68
Prevalence in White Nile State	White Nile	School-age children, general population, and fishermen	575,430	27.94%	22.96, 32.93

Schistosomiasis Prevalence Among Different Populations

**Figure 2:**
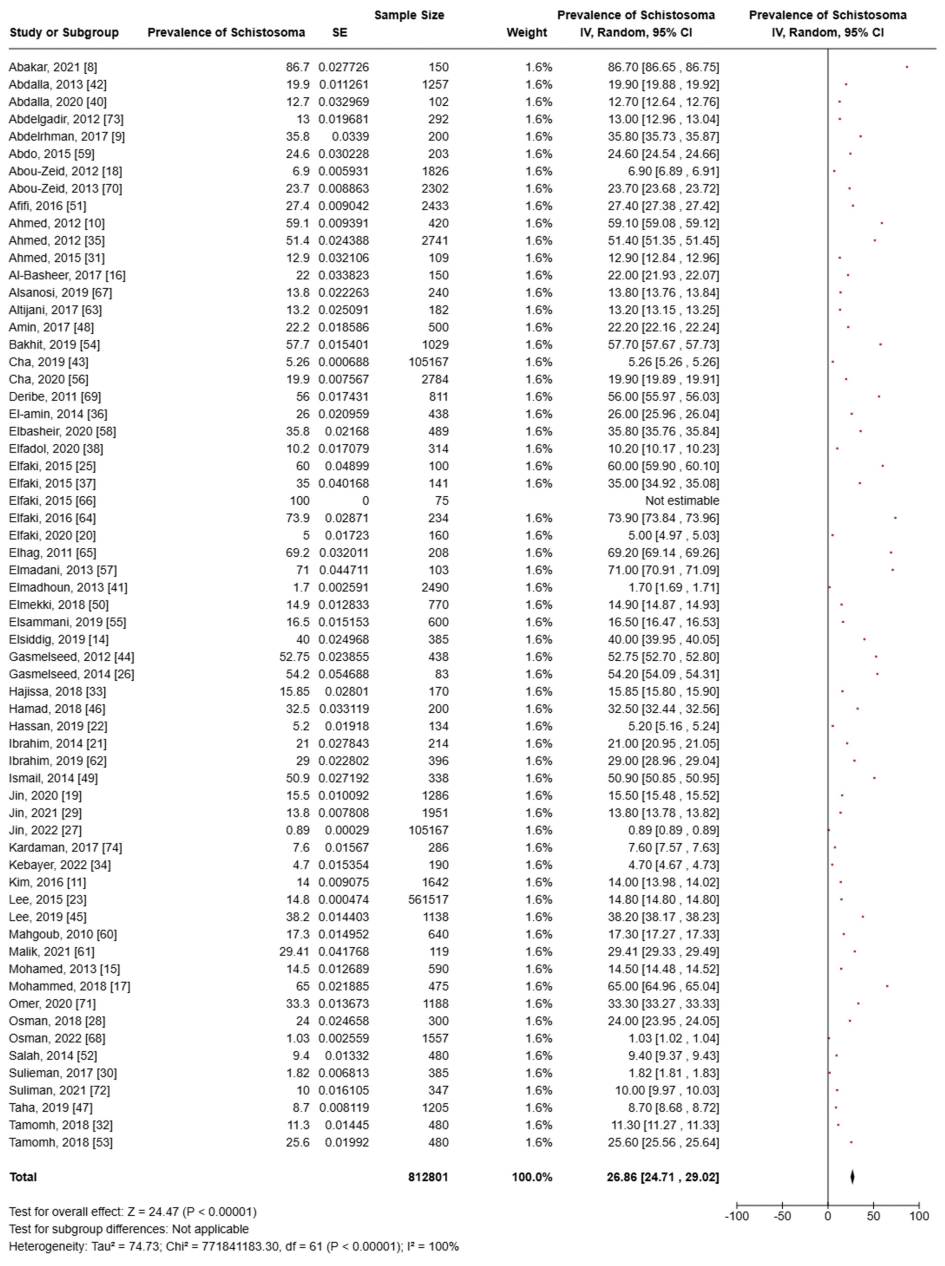
Meta-analysis of the prevalence of schistosomiasis among the participants of the included studies

Among 63 included studies to quantify the burden of the disease among the Sudanese population, despite the causative agent, and based on a total sample size of 812,801 participants of different populations as well as geographical locations, the pooled prevalence of schistosomiasis was 26.86% (95% confidence interval (CI): 24.71, 29.02). Heterogeneity was high (I^2^ = 100%) (Figure 2). The characteristics of all included studies are presented in Table 1.

Prevalence of S. haematobium

Forty-eight research articles determined the prevalence of *S. haematobium* [8,9,11,14,16-20,23,26,28-33,35,37,38,40-47,49-52,54-57,59,61-63,65,67-72,74]. Sixteen studies were conducted in White Nile State, 12 in Khartoum State, seven in Gezira State, four in River Nile State, three in Sennar State, two in southern Kordofan State, and one research article in each of Kassala, Gadarif, Northern State, South Darfur, and one in both Khartoum and Kassala States. Also, one study covered all 18 states in Sudan, representing a total sample size of 700,337 participants. Moreover, 38 articles were conducted among both genders, seven studies were conducted among males only, and the other three studies did not report the gender of their participants. Additionally, 39 studies were focused on the prevalence or risk factors among school-age children, 11 were toward the general population of different ages, two were conducted among patients and suspected patients, and one was conducted on fishermen. The pooled prevalence was 24.83% (95% CI: 22.75, 26.92). Heterogeneity was high (I^2^ = 100%).

Prevalence of S. mansoni

Twenty-eight research articles determined the prevalence of *S. mansoni* [10,15,18,20-23,25,33,34,36,43-45,48-51,53,55,58-60,62,64-66,70]. Seven studies were established in Kassala State, seven in Gezira State, and four in each of Khartoum and White Nile States. Moreover, three related articles were conducted in Sennar State, two in southern Kordofan State, one in both Khartoum and Kassala States, and one in all 18 states of Sudan, resulting in a total sample size of 685,133. Moreover, 27 articles recruited both genders, one study was conducted among males only, and one was conducted on females only. Furthermore, 20 studies were concerned with the prevalence or risk factors among school-age children. Additionally, nine studies were toward the general population, while one study was toward pregnant women. The pooled prevalence was 19.13% (95% CI: 18.70, 19.56). Heterogeneity was high (I^2^ = 100%).

Schistosoma Prevalence Among the General Population

Fifty-eight studies determined their participants as the general population (i.e., not being hospital outpatients or proposed to be at specific risk). Sixteen studies were conducted in White Nile, 11 in Khartoum, 10 in Gizera, eight in Kassala, four in River Nile, three in Sennar, two in Southern Kordofan, one in Gadarif, one in Northern State, and one in South Darfur State, resulting in a total sample size of 812,131. Fifty-one studies recruited both genders, six were among males, and three did not identify the gender of their participants. Age among the participants ranged from 0 to 99 years. The pooled prevalence was 25.75% (95% CI: 23.53, 27.97). Moreover, among the same population (general population), *S. haematobium* pooled prevalence was 22.84 (95% CI: 20.74, 24.95), while *S. mansoni* pooled prevalence was 19.37 (95% CI: 18.93, 19.82). Heterogeneity was high in all meta-analyses (I^2^ = 100%).

Schistosoma Prevalence Among School-Age Children

Schistosomiasis prevalence among school-age children was assessed in 45 included studies [9,10,14,16,17,19-22,26-30,32,33,35,36,38,41-44,46-50,52,53,55-58,60,62-64,67,68,70-72,74]. Twelve studies were conducted in White Nile, 10 in Khartoum, eight in Gezira, three in Sennar, four in River Nile, two in Kassala, one in Khartoum and Kassala, one in Gadarif, one in Southern Kordofan, and one in Northern State. Two studies covered all 18 states of Sudan, representing a total sample size of 240,228 participants. Thirty-eight research articles targeted both genders with participants of up to 20 years old. Four studies were conducted among males only, while the remaining three studies did not determine their participants' gender. The pooled prevalence was 24.46% (95% CI: 22.78, 26.13). Moreover, among the same population (school-age children), *S. haematobium* pooled prevalence was 22.37 (95% CI: 20.12, 24.63), while *S. mansoni* pooled prevalence was 18.62 (95% CI: 13.14, 24.11). Heterogeneity was high (I^2^ = 100%).

Schistosoma Prevalence in Khartoum State

*Schistosoma* prevalence in Khartoum State was investigated in 13 included studies [8,16,20,22,28,31,33,37,38,40,47,55,67]. The related studies were focused on school-age children, the general population, and suspected patients, resulting in a total sample size of 3,775 participants from two genders in the majority of studies. The pooled prevalence was 20.66% (95% CI: 11.74, 29.57). Moreover, among the same population (Khartoum States' residents), *S. haematobium* pooled prevalence was 21.55 (95% CI: 12.04, 31.07), while *S. mansoni* pooled prevalence was 2.47 (95% CI: 0.95, 4.00). Heterogeneity was high (I^2^ = 100%).

Schistosoma Prevalence in White Nile State

Seventeen included studies determined *Schistosoma* prevalence among White Nile State participants, representing a total sample size of 575,430 participants [9,11,14,17,19,23,29,32,42,45,49,53,54,56,61,63,72]. Sixteen studies were toward the general population, and one study was among fishermen. The majority of studies were toward both genders, one study was conducted among males only, and one study did not identify the age of their participants. The age of the participants ranged from 18 to 50 years. The pooled prevalence was 27.94% (95% CI: 22.96, 32.93). Moreover, among the same population (White Nile State residents), *S. haematobium* pooled prevalence was 27.49 (95% CI: 22.30, 32.69), while *S. mansoni* pooled prevalence was 8.77 (95% CI: 4.77, 12.78). Heterogeneity was high in all meta-analyses (I^2^ = 100%).

Schistosoma Prevalence in Gezira State

Eleven included studies determined *Schistosoma* prevalence among Gezira State participants, representing a total sample size of 5,712 participants [10,26,35,38,44,48,57,59,65,73,74]. Eight studies were toward school-age children or pregnant women. Seven studies recruited both genders; three studies were toward males, and one study was toward females of all ages. The pooled prevalence was 41% (95% CI: 26.72, 55,29). Moreover, among the same population (Gezira State residents), *S. haematobium* pooled prevalence was 38.59 (95% CI: 21.03, 56.14), while *S. mansoni* pooled prevalence was 25.85 (95% CI: 5.07, 46.63). Heterogeneity was high in all meta-analyses (I^2^ = 100%).

Schistosoma Prevalence in Kassala State

*Schistosoma* prevalence in Kassala State was examined in seven studies targeting the general population of school-age children, comprising a total sample size of 5,212 participants of various ages and both genders [15,25,34,51,60,64,66]. The pooled prevalence was found to be 32.97 (95% CI: 19.46, 46.47). Additionally, within the same population of Kassala State residents, it was not possible to determine the pooled prevalence for *S. haematobium* due to the inclusion of only one related study. However, the pooled prevalence for *S. mansoni* was calculated to be 30.33 (95% CI: 19.15, 41.51) based on the results from two included studies. Heterogeneity was high (I^2^ = 100%).

Schistosoma Prevalence in River Nile State

The prevalence of schistosomiasis among residents of River Nile State was evaluated in four included studies [30,41,46,71]. Three of these studies focused on school-age children, with a combined total sample size of 4,263 participants; only one study did not specify the gender of its participants. The pooled prevalence was determined to be 17.33% (95% CI: 6.44, 28.22). Furthermore, within the same population of River Nile State residents, the pooled prevalence for* S. haematobium* was 17.33% (95% CI: 6.44, 28.22). However, it was not possible to calculate the pooled prevalence for* S. mansoni* as this prevalence was not reported in any of the four included studies. Heterogeneity was high (I^2^ = 100%).

Schistosoma Prevalence in Sennar State

*Schistosoma* prevalence among residents of Sennar State was assessed in three included studies [21,58,62]. These studies focused on school-age children and comprised a total sample size of 1,099 participants of both genders. The pooled prevalence was calculated to be 28.60% (95% CI: 20.52, 36.68). Moreover, among the same population (Sennar State residents), *S. haematobium* pooled prevalence was available as *S. haematobium* prevalence was reported only in one related study, while* S. mansoni* pooled prevalence was 19.73 (95% CI: -2.86, 42.33) based on findings of two studies. Heterogeneity was high (I^2^ = 100%).

Sociocultural Factors Associated With Schistosomiasis

Sex: Sex was examined as a potential risk factor for schistosomiasis in 27 included studies. Participants comprised the general population and school-age children from all 18 states of Sudan. Among 67,531 male participants, the pooled odds ratio for male infection was 1.70 (95% CI: 1.39, 2.08), with a significant p-value of z = 5.22 (P < 0.00001). In contrast, there were 56,490 female participants from the same populations, with a pooled odds ratio for female infection of 0.59 (95% CI: 0.45, 0.76) and a significant p-value of z = 4.02 (P < 0.0001). Results are illustrated in Table 3.

**Table 3:** Summary of sociocultural risk factor estimates synthesized from the included studies OR: odds ratio, CI: confidence interval

Risk	Assessed in (state)	Assessed among	Total sample size	Pooled OR (95% CI)	Test for overall effect (Z score)
Male gender	All 18 states of Sudan	General population and school-age children	67,531	1.70 (1.39, 2.08)	5.22 (P < 0.00001)
Female gender	All 18 states of Sudan	General population and school-age children	56,490	0.59 (0.45, 0.76)	4.02 (P < 0.0001)
Illiteracy	Southern Kordofan, Kassala, North Kordofan White Nile, Gezira, and Khartoum	General population, pregnant women, and school-age children	1,496	0.26 (0.03, 2.07)	1.28 (P = 0.20)
Farming	All 18 states of Sudan	General population and school-age children	3,935	2.18 (1.12, 4.26)	2.29 (P = 0.02)
Fishing	All 18 states of Sudan	School-age children	652	1.51 (0.23, 9.81)	0.43 (P = 0.67)
Latrines	All 18 states of Sudan	School-age children and general population	81,940	0.62 (0.44, 0.88)	2.70 (P = 0.007)
No latrines	All 18 states of Sudan	School-age children and general population	24,301	1.62 (1.25, 2.09)	3.69 (P = 0.0002)
Canal and stream water source	Khartoum, South Kordofan, Kassala, North Kordofan, and White Nile	School-age children and general population	2,347	2.10 (1.07, 4.10)	2.17 (P = 0.03)
Donkey cart and tanker water source	Eastern Sudan, Kassala, Khartoum, and White Nile	School-age children and general population	167	0.59 (0.55, 0.64)	13.50 (P < 0.00001)
Pipe, tape, and hand pump water source	Khartoum, Eastern Sudan, South Kordofan, Kassala, North Kordofan, and White Nile	School-age children and general population	3,092	0.62 (0.34, 1.11)	1.61 (P = 0.11)
Swimming, playing, bathing, planting crops, and contact with water	Khartoum, South Kordofan, White Nile, Eastern Sudan, and River Nile	School-age children and general population	33,516	2.48 (1.81, 3.39)	5.67 (P < 0.00001)
No contact to water	All 18 states of Sudan	School-age children and general population	63,054	0.46 (0.28, 0.74)	3.15 (P = 0.002)

Education level: Illiteracy was examined as a possible risk factor for schistosomiasis across eight studies. The participants included individuals from the general population, pregnant women, and school-age children from Southern Kordofan, Kassala, North Kordofan, White Nile, Gezira, and Khartoum States, comprising a total sample size of 1,496. The pooled odds ratio for illiterate individuals being infected was 0.26 (95% CI: 0.03, 2.07); however, the p-value was not significant, with z = 1.28 (P = 0.20). Results are illustrated in Table 3.

Occupation: Farming occupation was examined as a possible risk factor for schistosomiasis across eight included studies. The participants included individuals from the general population and school-age children from all 18 states of Sudan. There were 3,935 farmers, and the pooled odds ratio of their infection was 2.18 (95% CI: 1.12, 4.26), with a significant p-value of z = 2.29 (P = 0.02). Moreover, fishing occupation was investigated among young 652 fishermen from different states; the pooled odds ratio of them being infected was 1.51 (95% CI: 0.23, 9.81), with an insignificant p-value of z = 0.43 (P = 0.67). The results are illustrated in Table 3.

Sanitation: The availability of latrines was assessed as a potential risk factor for schistosomiasis in nine studies. Participants included individuals from the general population and school-age children across all 18 states of Sudan. Among the 81,940 participants who reported having access to latrines, the pooled odds ratio for infection was 0.62 (95% CI: 0.44, 0.88), with a significant p-value of z = 2.70 (P = 0.007). In contrast, 24,301 participants from the same populations reported no access to latrines. The pooled odds ratio for this group being infected was 1.62 (95% CI: 1.25, 2.09), with a significant p-value of z = 3.69 (P = 0.0002). All results are presented in Table 3.

Water source: The use of canals and streams as water sources was examined as a potential risk factor for schistosomiasis in seven studies. Participants included individuals from the general population and school-age children in Khartoum, Southern Kordofan, Kassala, North Kordofan, and White Nile States, totaling 2,347 participants. The pooled odds ratio for infection in this group was 2.10 (95% CI: 1.07, 4.10), with a significant p-value of z = 2.17 (P = 0.03).

Additionally, the use of donkey carts (small tank vehicles pulled by donkeys, used for delivering water sourced mostly from wells in rural and semi-urban areas) and tankers was investigated in three studies involving participants from Eastern Sudan, Kassala, and White Nile States, including school-age children and the general population, with a total sample size of 167 participants. The pooled odds ratio for infection in this group was 0.59 (95% CI: 0.55, 0.64), with a significant p-value of z = 13.50 (P < 0.00001).

Furthermore, the use of pipes, taps, and hand pumps as water sources was studied in seven studies with participants from Khartoum, Eastern Sudan, Southern Kordofan, Kassala, North Kordofan, and White Nile States, encompassing both school-age children and the general population. The total sample size was 3,092 participants, and the pooled odds ratio for infection was 0.62 (95% CI: 0.34, 1.11), with an insignificant p-value of z = 1.61 (P = 0.11). All results are presented in Table 3.

Water contact: Contact with water through activities such as swimming, playing, or bathing was examined as a potential risk factor for schistosomiasis in 10 studies. Participants included school-age children and individuals from the general population in Khartoum, Southern Kordofan, White Nile, Eastern Sudan, and River Nile States, totaling 33,516 participants. The pooled odds ratio for infection in this group was 2.48 (95% CI: 1.81, 3.39), with a significant p-value of z = 5.67 (P < 0.00001).

Conversely, the absence of water contact was investigated across all 18 states of Sudan in five studies, involving school-age children and the general population, with a total sample size of 63,054 participants. The pooled odds ratio for this group was 0.46 (95% CI: 0.28, 0.74), with a significant p-value of z = 3.15 (P = 0.002). All results are presented in Table 3.

Discussion

To our knowledge, this review is the first attempt to evaluate the overall prevalence of schistosomiasis and its associated sociocultural risk factors in Sudan. The study utilized a thorough search across various published databases and employed a meticulous methodology for screening and selecting relevant studies.

In the current study, the pooled prevalence of schistosomiasis was 26.86% among 812,801 participants from all 18 states of Sudan. This finding is almost similar in comparison to a study done in Uganda (25.6%, 95% CI: 22.3, 29.0) [75]; however, a much lower estimate has been reported in the Philippines (8.4%, 95% CI: 3.5, 14.0) [76]. These differences may be attributed to social demographics and diagnostic protocols.

Moreover, the prevalence of *S. haematobium* was found to be 24.83% among 700,337 participants from different states. An even higher estimate has been reported in Zambia (35.5%) [77]. Furthermore, the prevalence of *S. mansoni* was 19.13% among 685,133 participants from all 18 states of Sudan. Higher estimates have been concluded in the literature as well. In neighboring Ethiopia, a prevalence of 26.3% was reported [78], while 34.9% was reported in Zambia [77]. These differences may be attributed to social demographics, study designs, and diagnostics protocols.

In regard to schistosomiasis prevalence among school-age children, the current study calculated the prevalence of schistosomiasis among 240,228 school-age children from different states as 24.46%. Higher estimates have been reported in neighboring Ethiopia (28.77%) [79] and Mozambique (52.8%) [80]. Such differences may be attributed to several factors, such as age-specific exposure patterns, school-based health programs, or social practices affecting water contact.

Moreover, *S. haematobium* prevalence among school-age children was 22.37. This finding is lower than the finding of a study conducted in Mozambique, which found the prevalence of* S. haematobium* to be 47% [80], as well as Zambia with 32.2% among the same population [77]. Nevertheless, the current finding is higher than the finding concluded in neighboring Kenya (14.8%) [81]. On the other hand, *S. mansoni* prevalence was 18.62 in the current study, which almost agrees with the finding concluded in a meta-analysis conducted among Zambians (18.1%) [77] but very much higher than the prevalence reported in Mozambique (1%) [80] and Kenya (1.2%) [81].

Furthermore, the current study found that males are linked to a higher rate of schistosomiasis infection in comparison with female gender. This finding is in alignment with a systematic review conducted earlier in Africa [82], as well as studies conducted in the Philippines and Ethiopia [76,79]. On the contrary, a study conducted in South Africa indicated that the female gender has a higher infection rate [83]. These differences may be attributed to variations in sociocultural characteristics among the study populations, such as gender-specific roles in water collection, which may expose males more frequently to contaminated water sources. Additionally, differing levels of access to healthcare and preventive measures, as well as variations in health-seeking behaviors between genders, could also contribute to these contradictory findings.

Regarding sanitation, the significant pooled odds ratio of participants being infected when latrines are unavailable was 1.62 (95% CI: 1.25, 2.09) in the current study. This finding is in agreement with the finding of a recent meta-analysis, as the authors stated that the odds ratio of schistosomiasis infection among participants with poor sanitation status is significantly increased [84].

Additionally, farming was indicated as significantly correlated to higher odds of schistosomiasis, which comes in agreement with the WHO's recent evidence [3]. Furthermore, being a fisherman was investigated as a potential risk factor in the current study. However, a 1.51 odds ratio was concluded with no significant difference. This finding opposed several reports [3,76,85,86]. Notably, the smaller sample size of fishermen in the current study (652 participants among three included studies) is to be considered when interpreting results.

Lastly, the current study indicated a significant association between water contact, such as bathing, washing clothes, collecting water for household use, fishing, and washing cars, and *Schistosoma* infection, which was previously reported in the literature [75].

Strengths and Limitations

The strengths of this review include the systematic identification and inclusion of relevant studies from 2010 to 2022. Additionally, a meta-analysis was conducted to generate pooled prevalence estimates from the included studies. Furthermore, a quality assessment was performed using criteria specifically designed to evaluate the quality of the selected studies.

Nevertheless, several limitations are to be considered when interpreting study results. Grey literature evidence was not assessed. Moreover, African journals that are not indexed in the screened databases were not considered for inclusion as well. Although all included studies are of good quality, several decent studies might have been missed. Furthermore, the heterogeneity was high in the meta-analysis conducted. Lastly, a potential limitation to acknowledge in this review is the impact of the current armed conflict in Sudan, which may influence the generalizability of the findings. Although the data included in the review was collected prior to the conflict, the sociopolitical instability, including the breakdown of healthcare infrastructure, interruptions in disease surveillance programs, and challenges in access to clean water and sanitation, may exacerbate the conditions for schistosomiasis transmission. The displacement of large populations and the potential for overcrowding in refugee camps or small villages further intensify the risk. Therefore, the findings should be interpreted with caution, considering the rapidly evolving situation, which may affect both disease transmission dynamics and access to preventive or therapeutic interventions.

## Conclusions

*Schistosoma haematobium* pooled prevalence was 24.83% (95% CI: 22.75, 26.92) among 700,337 participants tested, while *S. mansoni* pooled prevalence of 19.13% (95% CI: 18.70, 19.56) among 685,133 participants was found. Moreover, the highest *Schistosoma* prevalence (overall pooled prevalence: 41% (95% CI: 26.72, 55,29)) was found among Gezira State participants. Furthermore, farming, male sex, no presence of latrines, canal and stream water sources, and swimming, playing, or bathing in rivers and canals were found to be significantly associated with schistosomiasis infection. These findings serve as a cornerstone for designing targeted containment strategies and preventive measures, particularly in high-prevalence areas. Future interventions could focus on improving sanitation, promoting safe water practices, and raising awareness among vulnerable populations.

## Figures and Tables

**Figure 3:**
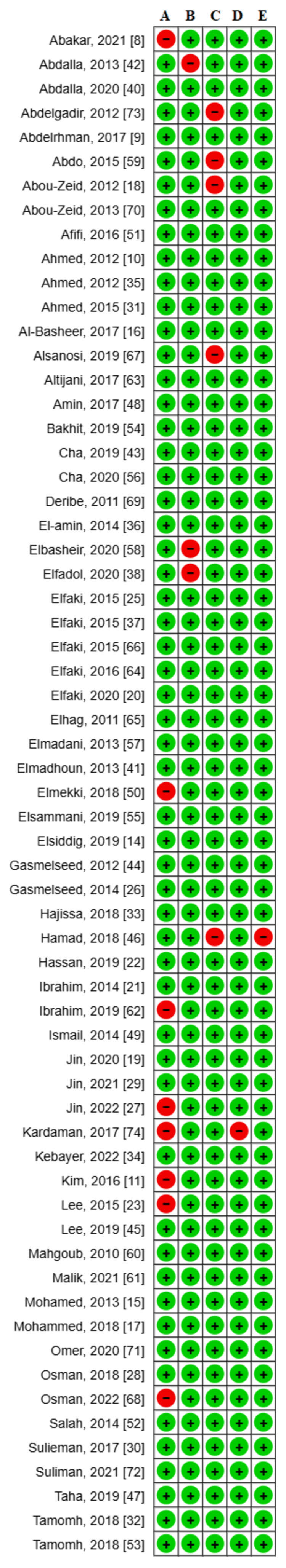
Risk of bias summary of the included studies A: Is the study objective clearly defined? B: Is the study sample completely determined? C: Is the study population clearly defined and specified? D: Is the methodology rigorous? E: Is the data analysis rigorous?

## Appendices

Table 4 shows the PRISMA checklist of the included studies.

**Table 4:** PRISMA checklist of the included studies PRISMA: Preferred Reporting Items for Systematic Reviews and Meta-Analyses, PICOS: participants, interventions, comparisons, outcomes, and study design

Section/topic	#	Checklist item	Reported on page #
Title			
Title	1	Identify the report as a systematic review, meta-analysis, or both.	1
Abstract			
Structured summary	2	Provide a structured summary including, as applicable: background; objectives; data sources; study eligibility criteria, participants, and interventions; study appraisal and synthesis methods; results; limitations; conclusions and implications of key findings; systematic review registration number.	1
Introduction			
Rationale	3	Describe the rationale for the review in the context of what is already known.	2
Objectives	4	Provide an explicit statement of questions being addressed with reference to PICOS.	3
Methods			
Protocol and registration	5	Indicate if a review protocol exists, if and where it can be accessed (e.g., Web address), and, if available, provide registration information including registration number.	NA
Eligibility criteria	6	Specify study characteristics (e.g., PICOS, length of follow-up) and report characteristics (e.g., years considered, language, publication status) used as criteria for eligibility, giving rationale.	3
Information sources	7	Describe all information sources (e.g., databases with dates of coverage, contact with study authors to identify additional studies) in the search and date last searched.	3
Search	8	Present full electronic search strategy for at least one database, including any limits used, such that it could be repeated.	3
Study selection	9	State the process for selecting studies (i.e., screening, eligibility, included in systematic review, and, if applicable, included in the meta-analysis).	3
Data collection process	10	Describe the method of data extraction from reports (e.g., piloted forms, independently, in duplicate) and any processes for obtaining and confirming data from investigators.	3
Data items	11	List and define all variables for which data were sought (e.g., PICOS, funding sources) and any assumptions and simplifications made.	4
Risk of bias in individual studies	12	Describe methods used for assessing the risk of bias of individual studies (including specification of whether this was done at the study or outcome level), and how this information is to be used in any data synthesis.	4
Summary measures	13	State the principal summary measures (e.g., risk ratio, difference in means).	4
Synthesis of results	14	Describe the methods of handling data and combining results of studies, if done, including measures of consistency (e.g., I^2^) for each meta-analysis.	5
Risk of bias across studies	15	Specify any assessment of risk of bias that may affect the cumulative evidence (e.g., publication bias, selective reporting within studies).	5
Additional analyses	16	Describe methods of additional analyses (e.g., sensitivity or subgroup analyses, meta-regression), if done, indicating which were pre-specified.	5
Results			
Study selection	17	Give numbers of studies screened, assessed for eligibility, and included in the review, with reasons for exclusions at each stage, ideally with a flow diagram.	6
Study characteristics	18	For each study, present characteristics for which data were extracted (e.g., study size, PICOS, follow-up period) and provide the citations.	8
Risk of bias within studies	19	Present data on the risk of bias of each study and, if available, any outcome level assessment (see Item 12).	15
Results of individual studies	20	For all outcomes considered (benefits or harms), present, for each study: (a) simple summary data for each intervention group (b) effect estimates and confidence intervals, ideally with a forest plot.	16
Synthesis of results	21	Present results of each meta-analysis done, including confidence intervals and measures of consistency.	16
Risk of bias across studies	22	Present results of any assessment of risk of bias across studies (see Item 15).	16
Additional analysis	23	Give results of additional analyses, if done (e.g., sensitivity or subgroup analyses, meta-regression (see Item 16)).	17
Discussion			
Summary of evidence	24	Summarize the main findings including the strength of evidence for each main outcome; consider their relevance to key groups (e.g., healthcare providers, users, and policymakers).	47
Limitations	25	Discuss limitations at the study and outcome level (e.g., risk of bias) and the review level (e.g., incomplete retrieval of identified research, reporting bias).	48
Conclusions	26	Provide a general interpretation of the results in the context of other evidence and implications for future research.	49
Funding			
Funding	27	Describe sources of funding for the systematic review and other support (e.g., supply of data), and the role of funders for the systematic review.	50

The quality assessment and risk of bias of the included studies are shown in Figure 3.
